# Effects of a 6-week on-court training program on the International Tennis Number (ITN) and a range of physical fitness characteristics in young tennis players

**DOI:** 10.3389/fspor.2024.1304073

**Published:** 2024-05-30

**Authors:** Jorge E. Morais, Bulent Kilit, Ersan Arslan, Yusuf Soylu, Henrique P. Neiva

**Affiliations:** ^1^ Department of Sports Sciences, Instituto Politécnico de Bragança, Bragança, Portugal; ^2^ Research Centre for Active Living and Wellbeing (LiveWell), Instituto Politécnico de Bragança, Bragança, Portugal; ^3^ Faculty of Sports Sciences, Tokat Gaziosmanpasa University, Tokat, Turkey; ^4^ Department of Sports Sciences, University of Beira Interior, Covilhã, Portugal; ^5^ Research Center in Sports, Health and Human Development (CIDESD), Covilhã, Portugal

**Keywords:** tennis, field tests, International Tennis Number (ITN), physical fitness, prediction

## Abstract

The study aimed to (i) verify the effects of an on-court training program on the International Tennis Number (ITN) of young tennis players, as well as on a set of change of direction, linear sprint, and maximal oxygen uptake (VO_2max_) variables, and (ii) identify the main predictors of ITN. The sample consisted of 20 young male tennis players (mean age, 13.62 ± 0.23 years). Players underwent a 6-week on-court training program. The ITN and a number of change of direction variables (T-drill and repeated sprint ability), linear sprint (5 m, 10 m, and 20 m distances), and VO_2max_ were measured. All variables improved significantly between the pre- and posttest (*p* < 0.001). The ITN (7.98 ± 6.06%, *d* = 0.82) and VO_2max_ (6.77 ± 1.21%, *d* = 1.53) showed the greatest relative improvement with moderate to large effect sizes. The hierarchical linear model retained the time (estimate = 18.90, *p* < 0.001) and the T-drill (estimate = −64.77, *p* < 0.001) as significant predictors of the ITN. This indicates that the ITN improved significantly over the 6-week training program and that the T-drill test was the best and most significant predictor. Coaches and researchers are encouraged to monitor the ITN along with other physical fitness and technical variables. They can also use the T-drill test to understand the ITN of their players.

## 1 Introduction

Tennis is a sport that is practiced worldwide in both recreational and competitive contexts. To improve performance, players must progressively develop and master technical skills essential for effective play during a tennis match (1). By doing so at an early age, tennis coaches or instructors can minimize a hypothetical skill mismatch between players, i.e., some players can meaningfully compete against less-skilled opponents and vice versa (2). Previous literature on tennis has focused on understanding the influence of specific body characteristics, biological maturation, or specific tests on youth tennis development (3–5). It has been noted that growth and maturation processes can have a significant impact on the development of young tennis players (3). This is especially true as each athlete is a unique individual, with a specific rate of development (6). For example, Fernandez-Fernandez et al. (4) conducted a study to examine maturational differences in the neuromuscular performance of young male and female tennis players. The assessment included a series of linear sprints, change of direction tests, and lower limb power. They reported that maturity level (such as peak height velocity) was a better parameter than chronological age when designing training programs specifically for youth tennis (4). Others aimed to understand the effects of specific field tests on player performance (7, 8). Overall, it was pointed out that certain fitness and technical tests should be used in youth tennis, as they provide useful information for the solid and sustainable development of the players.

As mentioned above, tennis players can practice or play against each other with an *a priori* meaningful mismatch between them. To better understand these skill differences, the International Tennis Federation has created the “World Tennis Number (WTN),” which is a scale from 40 to 1 (where 40 is a beginner and 1 is an elite player) that allows you to rank tennis players. According to the WTN website (https://worldtennisnumber.com/eng/faq), a special algorithm uses match results from 2016 onwards to calculate players' ranks. The more matches played, the more accurately the system can understand a player's skill level and rank them accordingly. The WTN aims to increase participation in tennis by allowing players of all skill levels to determine their individual level and thus make more solid progress. A recent study (9) reported that the WTN is a valid rating system for tennis. The authors measured the agreement between the WTN and the Universal Tennis Rating (UTR—considered the gold standard of tennis player rating systems) in 806 matches. There was a strong relationship between the WTN and the UTR, and both showed similar results for accuracy, sensitivity, and specificity in predicting match outcomes (9).

Notwithstanding, the ITF also has another rating protocol called the International Tennis Number (ITN), which consists of a scale of 10 to 1, with 10 being a novice player and 1 being an elite professional (10). The ITN is designed to be easily integrated into a standard coaching program and is also an ideal tool for use in tennis clubs. Unlike the WTN, the ITN consists of an on-court assessment that includes drills based on groundstroke depth, groundstroke accuracy, volley depth, serve, and mobility. Tennis is a physically and technically demanding sport (11). Thus, it can be argued that the ITN is more likely to better represent a player's level, as it is given based on the specific strokes and characteristics of tennis. For several years, the ITN has been used extensively by tennis coaches and researchers to classify player performance levels (12, 13). However, to the best of our knowledge, there is little evidence to understand the effects of on-court tennis training (OTT) on the ITN (14). Furthermore, no attempt was found to predict the ITN based on physical fitness variables that are related to tennis. As far as we know, (i) only two studies reported correlations between the ITN and variables related to physical fitness (15, 16), of which (ii) one study analyzed the effect of a training program on a specific feature of the ITN (13) and the other analyzed the effect of OTT and high-intensity interval training on the ITN (14). Thus, it can be argued that the literature lacks information on the ITN test, especially because it is a test used worldwide to classify or evaluate the level of players based on an on-court assessment. Based on the characteristics of the ITN assessment, one can argue that parameters related to the players’ physical fitness (namely related to their quickness) can have a meaningful effect on their ITN level and predict it. This can give coaches and practitioners information about which parameters the players should improve to get better scores in the ITN.

Therefore, this study aimed to (i) verify the effects of an on-court training program on the ITN of young tennis players, as well as on a set of change of direction, linear sprint, and maximal oxygen uptake (VO_2max_) variables and (ii) identify the main predictors of ITN. It was hypothesized that the on-court training program would significantly improve the players' ITN and all measured variables and the ITN would be predicted by the VO_2max_ simultaneously with another variable related to change of direction or linear sprint.

## 2 Methods

### 2.1 Participants

A total of 20 young male tennis players (age, 13.62 ± 0.23 years; height, 161.30 ± 8.27 cm; body mass, 51.95 ± 8.37 kg; maturity offset, −0.25 ± 0.26 years; peak height velocity, 13.86 ± 0.24 years) were recruited. All were right-handed tennis players with at least two years of experience in tennis training and competitions and were classified as Tier 3 athletes, i.e., highly trained athletes competing at the national or state level (17). The inclusion criteria required players to be uninjured and to have practiced regularly for the 6 months prior to data collection. Informed consent was obtained from parents or guardians and the players themselves. The study was carried out in accordance with the tenets of the Declaration of Helsinki and approved by the Local University Research Ethics Committee (47940-01).

### 2.2 Research design

The training intervention design consisted of 1 week of testing (pretest), 6 weeks of OTT, and 1 week of testing (posttest). The 6 weeks of OTT corresponded to the preparation period of the summer competitive season prior to the junior tennis season. During this period, players participated in three training sessions per week conducted on an indoor hard court. Heart rate (HR) measurements were continuously monitored using HR monitors (Polar V800, Polar Inc., Finland) to assess the intensity of the drills. Between the first week of the OTT and the sixth week, the average HR ranged between 169.6 ± 3.3 bpm and 174.5 ± 3.7 bpm, respectively. The players underwent a 10 min standardized warm-up period, which consisted of light aerobic activity (jogging), dynamic and passive stretching, and functional movements based on tennis-specific actions. Afterwards, they completed a 20 min set of on-court tennis-specific activities that included hitting against an opponent, focusing on consistency, and targeting specific areas of the court (e.g., cross or line shots, serve-return, groundstrokes, volleys). The players then underwent the on-court training program, which lasted 20–40 min. This was designed to improve start speed, acceleration, and speed endurance during tennis strokes, particularly the transition from forehand to backhand. The on-court training program incorporated maximal and submaximal running intensities and placed high demands on stroke quality as reported by others (14). The primary goal was to direct all shots to specific target areas on the baseline. In the on-court training program, various on-court tennis drills were performed using a racquet and ball. These drills followed procedures adapted from previous studies to ensure a structured approach to training (18, 19). The specific drill structure consisted of 2–3 sets of 5–6 repetitions, each lasting 30–60 s of work (10–20 strokes). There was a rest period of 30–60 s between repetitions and a rest period of 60–90 s between sets. The frequency of ball delivery was approximately one ball every 3 s. During the on-court training program, a coach positioned opposite the service boxes fed the balls to the players. The coach made sure that the balls were delivered at a consistent frequency and speed. Experienced coaches with level 3–4 certification from the Turkish Tennis Federation and 10–15 years of experience supervised the training program. At the end of the on-court training, the players performed individual interval training sessions without racquets, which lasted approximately 8–20 min. These sessions focused on explosive bursts to have the players exceed 85% of their maximum heart rate (HR_max_). At the end of the training session, the players underwent a 10 min cooldown period that included stretching exercises.

### 2.3 Anthropometric measurements and maturity

Body mass (in kilograms) was measured using a bioelectrical impedance analyzer (BC-418, Tanita, Tokyo). Players’ height (in cm) was measured using a stadiometer (Holtain Ltd., UK). Leg length was estimated as stature minus sitting height (which was measured with the stadiometer). A non-invasive technique was then used to assess the players’ maturity offset (20). Peak height velocity was calculated by subtracting the maturity offset value from the players' chronological age, as suggested by others (21).

### 2.4 The ITN test

**Figure 1:**
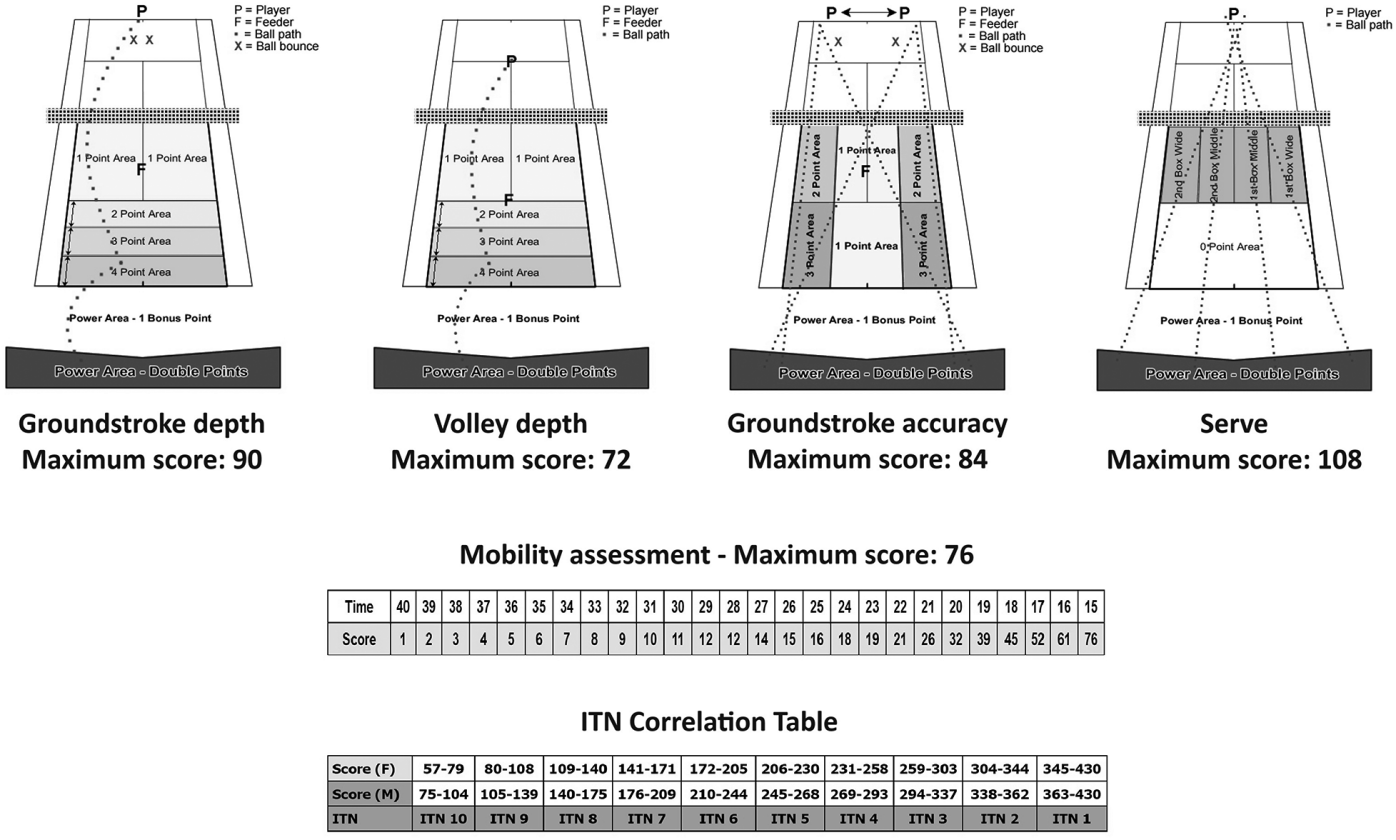
Illustration of the groundstroke depth, groundstroke accuracy, volley depth, and serve drills of the International Tennis Number (ITN) on-court assessment guide. Also shown are the mobility scores and the ITN correlation table, which allows the scores to be converted to the ITN.

The International Tennis Test (ITN) is an internationally recognized tennis rating that represents a player's general level of play. This is an objective on-court assessment tool based on tennis-specific tasks such as ball control, accuracy, and power. The ITN is a rating system where players are rated on a scale of 10 levels, from ITN 1 to ITN 10 (with 1 being the best). The ITN (in arbitrary units—a.u.) was performed on an indoor hard court to assess the player's technical skills. A ball machine (Tennis Tutor Plus, Sports Tutor, Inc., USA) was used to feed balls to the players under test. This on-court assessment consists of five technical elements, namely, groundstroke depth (maximum score, 90 points), groundstroke accuracy (maximum score, 84 points), volley depth (maximum score, 72 points), serve (maximum score, 108 points), and mobility (maximum score, 76 points), and was performed according to the International Tennis Federation guidelines for a maximum score of 430 points. The groundstroke depth assessment includes a power aspect and consists of 10 alternate forehands and backhands ground strokes. The groundstroke accuracy assessment also includes a power aspect and consists of six alternate forehands and backhands down the line and six alternate forehands and backhands crosscourt. The volley depth includes a power aspect and consists of eight alternate forehands and backhands volleys. The serve assessment includes a power aspect and 12 serves in total—3 serves in each target area. As for the mobility assessment, this measures the time it takes a player to pick up five tennis balls and return them individually to a specified zone. The scores from each element were added, and then the players' ITN number (i.e., tennis skill level) was reported based on an established normative table (10). Figure 1, which was taken from the official “ITN On-Court Assessment” guide, presents the specificities of the groundstroke depth, groundstroke accuracy, volley depth, and the serve drills (https://sonc.net/wp-content/uploads/2018/08/ITN-Assesment-Guide-levels-4-and-5.pdf). It also shows the mobility scores and the ITN correlation table, which allows the scores to be converted to the ITN.

### 2.5 Maximal oxygen uptake (VO_2max_)

The hit and turn tennis test was used to estimate the maximal oxygen uptake (VO_2max_, in ml/kg/min). After a typical 5 min warm-up, each player performed the test. This test is an acoustically controlled progressive on-court fitness test for tennis players. It was administrated according to the procedures proposed by (22). The maximal completed value was used to estimate the VO_2max_. After the test, the estimated VO_2max_ was calculated using the following formula (22):

(1) $$VO_{2\max}=33.0+(1.66\cdot \mathrm{HTTT})$$

where VO_2max_ is the maximal oxygen uptake (ml/kg/min) and HTTT is the player's final score on the hit and turn tennis test (a.u.).

### 2.6 Sprint tests

Each athlete performed a 20 m linear sprint test with 5 m, 10 m, and 20 m intervals (in seconds). The starting point was 70 cm behind the first pair of photocells (Witty, Microgate, Bolzano, Italy) marking the starting line. The participants were instructed to perform a maximal trial up to the 20 m timing gate. Four pairs of photocells were used (start line, 5 m, 10 m, and 20 m). The photocells were placed 0.4 m above the ground to minimize the effect of the hand swing when crossing the gate (23). They were activated upon crossing. The players performed two trials with a rest period of 120 s between trials. The best performance of the two sprints was used for further analysis.

### 2.7 T-drill agility test

The T-drill agility test (in seconds) is designed to assess agility performance. The test includes basic movements that are commonly performed in tennis training and matches. To complete the test, the players must sprint from a standing position to a cone placed 9.14 m away and then shuffle sideways to the left without crossing their feet to another cone placed 4.57 m away. After touching this cone, they shuffled to the right to a third cone placed 9.14 m away, shuffled sideways back to the center cone, and ran back to the starting point. Time was measured in seconds using a portable wireless photocell system (Witty, Microgate, Bolzano, Italy). The participants performed two trials with a rest period of 120 s in between. The best performance of the two sprints was used for further analysis.

### 2.8 Repeated sprint ability test (RSA)

The RSA test (in seconds) consisted of six repetitions of maximal 2 × 15 m shuttle sprints starting every 20 s (24). During the approximately 14 s recovery period between sprints, subjects were required to stand passively. Two seconds before the start of each sprint, the subjects were asked to assume the starting position as described for the 20 m sprints and wait for the start signal from a supervisor. Time was measured in seconds using a portable wireless photocell system (Witty, Microgate, Bolzano, Italy). The mean repeated sprint time (RSA_mean_) was used as a performance indicator (24). The participants performed two trials with a rest period of 120 s in between. The best performance of the two sprints was used for further analysis.

### 2.9 Statistical analysis

Descriptive data (mean ± SD) and the relative difference (%) were calculated. The paired samples *t*-test was used to verify the time effect (*p* < 0.05). The mean difference and 95% CI were used. Cohen's *d* was used to estimate the standardized effect size, which was considered to be (i) trivial, if 0 ≤ *d* < 0.20; (ii) small, if 0.20 ≤ *d* < 0.60; (iii) moderate, if 0.60 ≤ *d* < 1.20; (iv) large, if 1.20 ≤ *d* < 2.00; (v) very large, if 2.00 ≤ *d* < 4.00; and (vi) almost clear, if *d* ≥ 4.00 (25).

The hierarchical linear model (HLM) was used as a multilevel statistical procedure to identify the predictors of the ITN test. The ITN was chosen as the dependent variable because it strongly represents the movements and skills of a competitive tennis match. Two models were tested. The first model tested time (the difference between the pre- and posttest, i.e., training effect) as a predictor. This was done to understand if the ITN improved over time. The second model tested the remaining independent variables as predictors. Only significant predictors were included in the final model. Maximum likelihood estimation was calculated using the HLM7 software (26).

## 3 Results

Table 1 shows the descriptive statistics and the comparison of all measured variables between the pre- and posttest. The ITN and VO_2max_ showed the greatest relative improvement with 7.98 ± 6.06 and 6.77 ± 1.21%, respectively, between the pre- and posttest. All variables improved significantly (*p* < 0.001) between the pre- and posttest. This indicates that the OTT program tended to meaningfully elicit the ITN and all other variables. However, the T-drill test was the variable that improved with the largest effect size (*d* = 1.66).

**Table 1:** Descriptive statistics (mean ± SD) of the measured variables and comparison between the pre- and posttest.

	Mean ± SD			Paired samples comparison		Effect size
	Pretest	Posttest	Rel. diff. (%)	*t*-test (*p*-value)	Mean difference (95% CI)	*d* (descriptor)
ITN (a.u.)	215.90 ± 24.10	234.80 ± 21.93	7.98 ± 6.07	5.40 (<0.001)	18.90 (11.58–26.22)	0.82 (moderate)
VO_2max_ (ml/kg/min)	45.33 ± 2.05	48.63 ± 2.25	6.77 ± 1.21	22.46 (<0.001)	3.30 (2.99–3.61)	1.53 (large)
5 m sprint (m)	1.14 ± 0.05	1.08 ± 0.05	6.32 ± 3.10	9.28 (<0.001)	0.07 (0.05–0.08)	1.20 (large)
10 m sprint (m)	2.16 ± 0.10	2.06 ± 0.08	4.66 ± 1.52	12.90 (<0.001)	0.10 (0.08–0.11)	1.10 (moderate)
20 m sprint (m)	3.61 ± 0.21	3.45 ± 0.21	4.66 ± 1.38	14.90 (<0.001)	0.16 (0.14–0.18)	0.76 (moderate)
RSA_mean_ (s)	6.57 ± 0.11	6.37 ± 0.15	3.24 ± 1.88	7.64 (<0.001)	0.20 (0.15–0.26)	1.52 (large)
T-drill (s)	12.57 ± 0.16	12.26 ± 0.21	2.55 ± 0.97	11.72 (<0.001)	0.31 (0.26–0.37)	1.66 (large)

Rel. diff., relative difference; ITN, international tennis number; VO_2max_, maximal oxygen uptake; RSA_mean_, mean repeated sprint ability; 95% CI, 95% confidence intervals; *d*, Cohen's *d* effect size.

Table 2 presents the multilevel modeling data. Time (estimate = 18.90, *p* < 0.001, 95% CI = 12.22–25.58) and T-drill (estimate = −64.77, *p* < 0.001, 95% CI = −82.04 to −47.50) remained significant predictors of ITN. For each unit increase in T-drill (in seconds), the ITN decreased by 64.77 points. Thus, lower scores on the T-drill (better performance) would lead to better performance on the ITN.

**Table 2:** Hierarchical linear model coefficients with 95% CI.

	Estimate	SE	95% CI	*p*-value
Time	18.90	3.41	12.22 to 25.58	<0.001
T-drill (s)	−64.77	8.81	−82.04 to −47.50	<0.001

The following equation can be used to calculate the players' ITN based on the T-drill test:

(2) ITN=1029.41−(64.77⋅T−drill)

## 4 Discussion

This study aimed to (i) verify the effects of an on-court training program on the ITN of young tennis players, as well as on a set of change of direction, linear sprint, and VO_2max_ variables, and (ii) identify the main predictors of the ITN. The main findings indicate that the ITN and the remaining variables improved significantly with the on-court training program. On the other hand, the second hypothesis of this study was partially accepted, i.e., only one variable related to change of direction (T-drill) was retained as a main predictor of the ITN.

Regarding the use of variables to measure physical fitness in tennis, a review study on this topic showed that there is a general disagreement in the scientific community regarding the most important physical characteristics and useful tests in tennis (8). The authors summarized the different physical tests recommended and used by practitioners, sport scientists, and institutions (national tennis federations) (8). Therefore, this experimental design included some of the tests indicated as the most appropriate for tennis players (e.g., linear sprints and the hit and turn tennis test—this one to estimate VO_2max_). In addition, based on more recent evidence, the T-drill test and the RSA were also included, as they also represent tennis movements (27, 28).

Literature can be found on the effects of training programs on variables related to the physical fitness of tennis players (12, 14, 29). However, different results have been found in age groups like those in this study. For example, a study by Fernandez-Fernandez et al. (29) reported that a sport-specific drill training alone failed to promote meaningful improvements in linear sprint, lower limb strength, and agility, with only trivial to small effect sizes. This training design only produced meaningful improvements in oxygen uptake with a moderate effect size. However, when compared to a combined sport-specific drill training and high-intensity training, the latter training design promoted greater improvements (based on the relative difference) in all of these parameters than sport-specific drill training alone (29). On the other hand, others found that both OTT alone and high-intensity interval training programs were able to induce linear sprint, lower limb strength, agility, and oxygen uptake in fourteen-year-old players (14). The differences between groups were only found in the 400 m time and agility tests. It can be argued that the main factor responsible for such an improvement in both training designs was that the OTT had a specific focus on different types of OTT drills. These were based on speed and acceleration at maximal and submaximal intensities, mostly involving the anaerobic system during short- and high-intensity efforts (14). The current data showed similar results, where the OTT program alone was able to promote significant gains in all measured variables, confirming the first hypothesis of this study. OTT typically consists of a variety of activities designed to improve different aspects of a player's game (i.e., technical, tactical, and mental) (30). However, in the present study, the OTT was also designed to improve the players' physical fitness and technical skills simultaneously. Therefore, the 6-week OTT alone significantly improved the players' physical fitness parameters with moderate to large effect sizes. Notwithstanding, it should be mentioned that these physical fitness variables can be improved even more when high-intensity interval or repetitive sprint training programs are added to the sport-specific training or OTT programs (12).

Only one study was found regarding the objective effects of a specific training program on the ITN (14). Contrary to what was found for the physical fitness variables, the ITN improved significantly in players who underwent an OTT alone rather than a high-intensity interval training (14). As previously reported, OTTs focus on a variety of topics related to player performance rather than just physical fitness, as may be the case with high-intensity interval training. Therefore, if players spend more time performing or training their technical skills, they are more likely to improve their ITN. Another study also showed the effect of rope jumping training on a specific characteristic of the ITN, i.e., accuracy and depth of impact in the stability test (13). The authors found that the experimental group significantly improved both the accuracy and depth in the hit stability test (13). In this case, it appears that this specific physical fitness task allowed the players to improve some parameters of the ITN. Thus, based on the scarce evidence on this topic, it can only be argued that there may be specific physical fitness tasks or drills that can improve the ITN of players. As this test is widely used by coaches and several federations to classify the level of their players based on a number of technical and physical characteristics, it was expected that there would be some evidence on this topic. On the contrary, it was only found that studies with a pre- vs. posttest mainly used the ITN to classify their sample, but not to understand the underlying effects of the ITN (31, 32).

As mentioned above, the ITN test is a worldwide tool for coaches to understand and rank the skill level of their players. In the present approach to the problem, it was decided to use only motion-based variables to predict the ITN. That is, since the ITN is a motion-based test (i.e., it is performed based on the player's displacement) that aims to simulate a tennis point, the variables included are also motion-based. The HLM statistical procedure was used to identify the predictors of the ITN test. Time and the T-drill were retained as significant predictors. Time as a significant predictor confirmed the results of the *t*-test, where the on-court training program allowed a significant improvement in the ITN. As for the T-drill test, lower scores (better performances) on this test led to higher scores (better performances) on the ITN. It should be noted that although the ITN evaluates the technical skills of the players, these can also be related to physical fitness. It has been shown that players with higher physical fitness are more likely to have better skills (8, 33).

Nevertheless, there is a lack of evidence in the literature regarding the relationship between physical fitness variables and the ITN. As mentioned above, most studies on tennis that include physical and technical characteristics do not include the ITN except for sample classification (12, 34). Nevertheless, one study aimed to understand the relationship between strength and power, muscle stiffness, stroke velocity, and the ITN in junior tennis players (15). The authors found that the pectoralis major stiffness and maximal isometric wrist flexion strength were significantly and directly correlated with the ITN (i.e., greater stiffness was correlated with smaller ITN—poorer performance). It has been argued that higher levels of stiffness, especially in the upper body, may have some negative influence on the ITN by interfering with speed production (15). Others noted that the ITN in young children (8–10 years old) was significantly and directly correlated with the standing long jump, that is, greater distances were correlated with better performance on the ITN (16). However, none of these predicted the ITN. The present results suggest that the ITN can be predicted based on a faster field test such as the T-drill, which represents tennis movements. It has been reported that for some athletes, gains in strength and power may not benefit agility skills (35). It can be argued that, depending on player characteristics (in a holistic perspective), excessive lower limb strength and power indices may impair some players' agility. Therefore, dynamic tests, such as the T-drill, may be more representative of the ITN rather than static tests.

The main findings indicate that OTT programs alone can significantly improve the physical fitness characteristics and ITN of tennis players. However, this improvement may be related to the specificity of the OTT content. The ITN is a worldwide test for understanding and ranking the level of the players. Therefore, it can be argued that whenever coaches and researchers want to understand the effects of a given program on physical fitness and technical variables, they should also monitor the ITN. This may provide a deeper insight into the effects of such variables on the ITN. In addition, physical characteristics (specifically the T-drill test) were able to predict the ITN. Thus, coaches and players can use this faster test to meaningfully determine a player's ITN. Furthermore, based on a training or more professional context, ITN monitoring can also help coaches understand the level of their players and cluster them to design more appropriate training programs (36). The main limitations are that (i) these results are only applicable to this age group; (ii) there are other dynamic tests (physical or technical) that may help in the prediction of ITN; and (iii) the VO_2max_ was estimated rather than directly measured. Regarding the estimation of VO_2max_, it should be mentioned that it is based on a specific field test in tennis (i.e., the hit and turn tennis) (22), which has been validated for both sexes and several age groups. For the specific age group used in the current study, a moderate correlation was found between the measured and the estimated VO_2max_ with an absolute marginal difference (measured VO_2max_ = 55.0 ± 4.0 ml/kg/min; estimated VO_2max_ = 54.3 ± 2.0 ml/kg/min) (22). Thus, this is a valid test that is commonly used by tennis researchers and coaches to measure the aerobic capacity of players. Finally, future studies could focus on understanding these relationships in other age groups and test other physical or technical parameters that can be related to the ITN. Given the paucity of evidence on this topic and the importance of measuring the ITN, researchers might also aim to understand the effects of different training designs (OTT or others) on ITN performance.

## 5 Conclusions

On-court training programs based on tennis-specific skills can significantly improve physical fitness variables and ITN. Dynamic tests, particularly the T-drill test, can predict the ITN, with better T-drill scores being associated with better ITN performance. Tennis coaches and researchers should be encouraged to monitor the ITN simultaneously with physical and technical variables, as this can provide a deeper insight into the level of the players.

## Data Availability

The raw data supporting the conclusions of this article will be made available by the authors, without undue reservation.
